# What is the best histopathological classification for celiac disease? Does it matter? 

**Published:** 2015

**Authors:** Amado Salvador Peña

**Affiliations:** *Laboratory of Immunogenetics, Department of Medical Microbiology and Infection Control, Vrije Universiteit Medical Center (VUmc), Amsterdam, the Netherlands*

The rebuttal of Oberhuber’s sub-division of Marsh III type in celiac disease (1) and its subsequent correspondence in the past and present issues of GHFBB allow us to review the current thinking on the histopathological classification of celiac disease. In the Definition of Medicine, Avicenna argues: -“*While some divide medicine into a theoretical and a practical [applied] science, others may assume that it is only theoretical because they see it as a pure science. But, in truth, every science has both a theoretical and a practical side*”. Thus, if you are working in your area in a community hospital without a dedicated pathologist to celiac disease, you could use either the classification made by Corazza & Villanacci (2) or by Ensari (3). Both classifications are practical and have proven to be useful with good specificity and sensitivity. Both the Oberhuber and the Corazza & Villanacci classifications allow discriminating latent celiac disease from patients with normal mucosa. Their classification may help in identifying and treating patients at an early stage (4). 

It is crucial to state that several biopsy samples of the duodenum and one from the bulbus duodeni should be taken. The specimens should be properly oriented to be able to assess the correct histopathological features. These classifications were developed to avoid the morphometric techniques, which at present are not used in routine histological laboratories. Marsh used morphometry in his extensive and now classical studies of the small intestine in celiac disease (5-8). Judging by the citation index the two classifications (76 and 33 respectively) cannot compete with the 1283 citations of the Marsh article of 1992 and the by now (September 2015) 656 citations of the Oberhuber et al article of 1999 even considering the difference in years of the publications. 

The morphometric techniques are time-consuming and unpractical when technical facilities are not available. Ensari wanted to avoid the subjective element of the subdivision of the Type III lesions which were introduced by both Rostami et al (9, 10) and Oberhuber et al (11). When comparing the interobserver reproducibility of the “Marsh-Oberhuber” classification versus the one used by Corazza & Villanacci it was shown that the overall mean kappa value was 0.35 (fair) for the “Marsh-Oberhuber” classification and 0.55 (moderate) for the Corazza & Villanacci classification (12). 

Based on a sophisticated comparison between scanning electron microscopy studies and histological assessment, Marsh et al found that the Oberhuber's revisions of Marsh III into three sub-categories (IIIa, IIIb, IIIc) are misinterpretations of the histological appearances of flattened mucosae. Therefore, Marsh et al, concluded that histopathologists when classifying celiac mucosae should avoid the IIIa, IIIb, IIIc subcategories, because it does not add to the diagnostic or prognostic value of his original classification (1). However, the difference in positivity of endomysium antibodies (EMA) of the Marsh IIIa and Marsh IIIc groups were significant (P<5= 0.013). The differences found in Marsh IIIa and Marsh IIIb, compared with Marsh IIIc for antibody positivity, were also significant (P<0.01) (10). The Rostami et al study (10) demonstrated the value of the subclassification of Marsh III in relation to serology when an expert pathologist is consistent with the use of his own criteria. Practical applications are as valuable as theoretical ones (Avicenna).

According to Ensari (3) a problem arising from the Marsh classification is the cut-off for the number of intra-epithelial lymphocytes (IELs) which were counted with respect to a standard test area of 104 mm^2^ of the muscularis mucosae in order to define the absolute numbers in a 3-dimensional fashion (6, 7). Ensari also stated that the distribution pattern of IELs within the epithelium is more valuable than the actual counts. Recently, Marsh has pointed out: -how can there be a “cut-off” between a so-called “normal” and a “celiac” lesion when in fact the IELs are graded characteristics (8). The Oberhuber classification did not resolve the issue at the time of their publication. Possibly the 40 IELs per 100 enterocytes used as a cut-off criterium for normality was derived from jejunal mucosal biopsies. The intestinal biopsies were previously performed with biopsy-capsule instruments instead of endoscopy. Also the different section thickness of the specimens probably interfered with the correct counting of the IELs. The upper limit of IELs is 25 per 100 enterocytes in the duodenal mucosa (3). The use of immunochemistry, in particular CD3 stained sections, allows a more precise evaluation (13). Table 1 summarizes the main characteristics of the classifications with some suggested modifications. The classifications are practical although a low reproducibility has been reported in multicenter studies. All classifications depend on the preparation of the biopsy specimens and their correct orientation to avoid allocating the patients in the wrong category (14). For example, in a multicenter study in Europe, it was found that the quality of the biopsy specimens was not acceptable in 29 (10.7%) of 271 cases. A reliable judgment could not be made, mainly due to the poor orientation of the biopsy samples. The primary clinical diagnosis and the second classification of the biopsy specimens were divergent in nine cases, and one patient was initially enrolled in the wrong group (15). Notice in the table that the most recent actualization incorporating “Type 0” in celiac disease originates from recent concepts in microscopic enteritis (16, 17) and as Marsh stated: -“*genes and prolamins are logically anterior to intestinal damage*”(8). Since “Microscopic Enteritis” may be the most common histological finding in celiac disease (18-20) the original classification of Marsh from 1992 (5) has been updated in recent publications in 2015 (17). Probably this is the most up-to-date classification of celiac disease in academic medicine. Even with this new classification, new tools for the diagnosis of celiac disease may become necessary in the following clinical situations: 1) HLA-DQ2, HLA-DQ8, occasional HLA-DQ2.2, HLA-DQA1*05 and possibly HLA-DQ9.3 positive Caucasian individuals on a self-prescribed gluten-free diet; 2) Patients with seronegative villous atrophy; 3) HLA-DQ2 and/or DQ8 positive patients with lymphocytic enteritis and either positive (often with low/borderline titers increasing the risk of false positives) or negative celiac specific serological tests; 4) Monitoring gluten reactivity in latent or potential celiac disease; 5) First-degree relatives of patients with celiac disease with the highest risk of developing the disease (21).

How important is the focus on intestinal histopathology in celiac disease when -“*it is time to change the historical dogma that defines histology as the gold standard for the detection of celiac disease*” (22). Are these classifications reliable enough to the new phase of celiac disease? For the first time in history in this disease, there is a development of drugs which are in different stages of research and development (23). Registration trials and regulatory agencies for new drugs or the development of a therapeutic vaccine will need reliable “trial endpoints”. For this new era of celiac disease, the morphometric techniques cannot be discarded as unpractical. Classifications based on objective quantitative morphological parameters such as measurements of height-to-crypt-depth ratio and inflammatory variables such as the density of IEL with a proper protocol are welcome (24). 

**Table 1:** Histopathological classifications of celiac disease

Marsh 1992 and Rostami et al. 2015 (5, 8, 16, 17)	Rostami et al 1998, 1999 (9, 10)	Oberhuber et al. 1999 (11)	Corazza & Villanacci 2005 (2)	Ensari 2010 (3)
**Type 0: **Microscopic enteritis; normal villi with pathological increase of T lymphocytes, alteration of enterocytes, shortening of microvilli and increased α/β/γ/δ T cell receptors				
**Type 1: **Microscopic enteritis**:** increased IEL count (> 20 IEL/100 enterocytes)	**Marsh I**: normal villous epithelium > 30 IEL per 100 enterocytes	**Type 1** Infiltrative lesion	**Grade A** No atrophy, normal villous architecture with or without crypt hyperplasia and ≥25 IELs/100 enterocytes	**Type 1** Normal villi with IE lymphocytosis
**Type 2** Microscopic enteritis increased IEL count (> 20 IEL/100 enterocytes) and crypt hyperplasia)	**Marsh II**: enlarged crypts and influx of inflammatory cells	**Type 2** Crypt hyperplasia	**Grade A**	**Type 1**
**Type 3** Villus effacement and crypt hyperplasia	**Marsh IIIa**: (partial VA) shortened blunt villi, infiltration IEL and hyperplastic crypts	**Type 3A: Partial**	**Grade** **B1** villous-crypt ratio <3:1 IEL count of >25/100 enterocytes**	**Type 2 **Shortened villi (<3:1 or <2:1 in bulbus) with IE lymphocytosis and crypt hyperplasia
	Marsh IIIb (subtotal VA) Recognizable atrophic villi, inflammatory cells and enlarged crypts	**Type 3B: Subtotal**	**Grade** **B1**	**Type 2**
	**Marsh IIIc** :(total villous atrophy) total absence of villi, severe atrophic, hyperplastic, infiltrative lesion	**Type 3C: Total**	**Grade B2** Completely flat atrophic mucosa, no observable villi and ≥25 IELs/100 enterocytes	**Type 3** Completely flat mucosa with IE lymphocytosis and crypt hyperplasia
**Type 4** Destructive lesion	Not considered	**Type 4** Destructive lesion	Not considered	Not considered

Modified from Fernández-Bañares et al. (3, 20).

* in jejunum and different section thickness in the past,

** upper limit of normal in duodenal mucosa. IEL= intraepithelial lymphocyte

We can conclude that the classification we use in celiac disease matters. We will have to reassess the histological morphometry standards established in the last century by Rubin, Marsh, Corazza, Ensari and other clinical scientists. However with new tools, such as quantitating immunological parameters to determine the presence of IgA tTG local intestinal antibodies, the IELs expressing gamma/delta T cell receptor, and probably other parameters, by using the latest immunological concepts and techniques. As Lonardi et al (25) have observed, TCRgamma coupled with CD3 staining, may represent an additional tool to recognize cases of latent/potential celiac disease when serology and clinical data are not conclusive or when the histological diagnosis remains equivocal. The primary endpoint for clinical trials could be the use of histological morphometry complemented by markers. These new approaches and the new mucosal and serological biomarkers in development, combined with gene expression at the mucosal intestinal level are already on the horizon and will facilitate the analysis of effectiveness of drugs for celiac disease
